# Experimental Setting for Applying Mechanical Stimuli to Study the Endothelial Response of Ex Vivo Vessels under Realistic Pathophysiological Environments

**DOI:** 10.3390/life11070671

**Published:** 2021-07-08

**Authors:** Ana Osuna, Anna Ulldemolins, Hector Sanz-Fraile, Jorge Otero, Núria Farré, Ramon Farré, Isaac Almendros

**Affiliations:** 1 Unitat de Biofísica i Bioenginyeria, Facultat de Medicina i Ciències de la Salut, Universitat de Barcelona, 08036 Barcelona, Spain; anaosuna412@gmail.com (A.O.); anna.ulldemolins@ub.edu (A.U.); hector.sanz.fraile@hotmail.com (H.S.-F.); jordi.otero@gmail.com (J.O.); rfarre@ub.edu (R.F.); 2 CIBER de Enfermedades Respiratorias, 28029 Madrid, Spain; 3 Heart Diseases Biomedical Research Group, IMIM (Hospital del Mar Medical Research Institute), 08003 Barcelona, Spain; nfarrelopez@psmar.cat; 4 Department of Medicine, Universitat Autònoma de Barcelona, 08193 Bellaterra, Spain; 5 Heart Failure Unit, Department of Cardiology, Hospital del Mar, 08003 Barcelona, Spain; 6 Institut d’Investigacions Biomèdiques August Pi Sunyer, 08036 Barcelona, Spain

**Keywords:** bioreactor, vessel mechanics, experimental physiology, mechanical stimuli, hypoxia, open-source hardware

## Abstract

This paper describes the design, construction and testing of an experimental setting, making it possible to study the endothelium under different pathophysiological conditions. This novel experimental approach allows the application of the following stimuli to an ex vivo vessel in a physiological bath: (a) a realistic intravascular pressure waveform defined by the user; (b) shear stress in the endothelial layer since, in addition to the pressure waveform, the flow through the vessel can be independently controlled by the user; (c) conditions of hypo/hyperoxia and hypo/hypercapnia in an intravascular circulating medium. These stimuli can be applied alone or in different combinations to study possible synergistic or antagonistic effects. The setting performance is illustrated by a proof of concept in an ex vivo rabbit aorta. The experimental setting is easy to build by using very low-cost materials widely available. Online Supplement files provide all the technical information (e.g., circuits, codes, 3D printer drivers) following an open-source hardware approach for free replication.

## 1. Introduction

Alterations in the state of cellular physioxia are common in different pathophysiological conditions. For example, hypoxia is a normal circumstance at high altitude and a pathological condition in a number of highly prevalent chronic diseases, such as pulmonary airway obstruction, obesity/hypoventilation syndrome, sleep apnea or heart failure, and in acute severe conditions such as during mechanical ventilation of patients with respiratory distress syndrome [1,2,3,4]. Since, in many cases mechanical ventilation must be carried out with an enriched oxygen concentration, hyperoxic challenge is also a risk in many patients in intensive care units [5,6]. Any alteration of physioxia runs the risk of altering the normal balance of oxidative stress, with associated harmful consequences [6]. Given the importance of investigating how cells and tissues are affected by hypoxia/hyperoxia, well-designed experimental settings are required to monitor the partial pressure of oxygen in culture dishes or in baths for ex vivo tissue preparations [7,8,9,10,11,12]. Importantly, in addition to controlling oxygen, the study of cells/tissues of organs physiologically subjected to mechanical stress (e.g., lung, heart, vessels, tendons) also requires the use of experimental settings capable of applying realistic mechanical stimuli, since solid evidence on mechanobiology indicates that mechanical stress considerably modulates cell/tissue function [13,14].

Alterations in the endothelial cell layer characterize several diseases with high prevalence and impact in public health [15]. Consequently, considerable research efforts are devoted to investigating the basic mechanisms involved in endothelial dysfunction and to developing preventive and therapeutic drugs. As it is usual when studying any organ or tissue, research on endothelial dysfunctions is carried out by means of the classical multilevel triad: cell cultures, animal models and human studies. Interestingly, however, and at variance with other tissues, the endothelium can also be subjected to ex vivo research in a relatively easy way since blood vessels can be isolated and kept acceptably functional inside baths with suitable internal and external media. Ex vivo vessel preparation is an interesting level of research (which is intermediate between cell culture and animal model) since it allows to investigate the endothelial layer in its natural substrate—the vessel wall—instead of under the artificial conditions of 2D cultured cells seeded on rigid plates. Although a drawback of ex vivo vessels is that they are isolated from the physiological systemic interactions from other tissues and organs, they present the advantage of permitting to study the whole endothelium under well controlled experimental conditions.

However, ex vivo investigation of endothelial pathophysiology under optimal realistic conditions requires the use of settings allowing to reproduce the different stimuli experienced by the endothelium in vivo. Realistically mimicking the natural environment requires that—in addition of using suitable maintenance media with/out mediators (e.g., inflammatory agents) or drugs—the ex vivo vessel, and, hence, its endothelial layer, is subjected to the same mechanical stimuli as in vivo. Specifically, compression and cyclic stretch caused by pulsatile blood pressure (increased in systemic arterial or pulmonary hypertensions) [16,17] and shear stress caused on endothelial cells by blood circulation with potentially abnormal viscosity [18]. Moreover, an optimal setting for ex vivo vessels should be able to circulate an intravascular medium with well controlled partial pressures of O_2_ and CO_2,_ since in many relevant diseases encompassing endothelial dysfunction the patient experiences abnormal levels of blood gases. For instance, hypoxia/hypercapnia in chronic obstructive pulmonary disease and heart failure [19,20], hyperoxia events in patients mechanically ventilated with a high-oxygen fraction [21] or intermittent hypoxia/hypercapnia in patients with sleep apnea [22].

Unfortunately, it is not easy to find or to assemble an affordable experimental setting which allows a controlled application of such a combination of realistic stimuli to study the endothelium ex vivo. Moreover, the examples available in the literature briefly describe the experimental setting but do not provide enough instructions for easily replicating and assembling different devices which are usually complex and expensive [22,23,24] The aim of the work presented herein was to design and test a performant compact setting for this application which is easy to build and cheap. Most importantly, we followed the open-source hardware approach [25,26] consisting of placing all the technical information available to the community for free replication. This approach is useful to facilitate spreading of medical devices [27,28,29,30] and scientific settings [25]. Accordingly, all the technical information required to assemble the developed experimental setting (electronic circuits, sensors, mechanical pieces and microcontroller) is freely provided as Supplementary Materials. Hence, schematics and ready-to-use files are available to the scientific community, allowing researchers to implement and easily use the device, thereby facilitating endothelial research.

## 2. Materials and Methods

**Figure 1:**
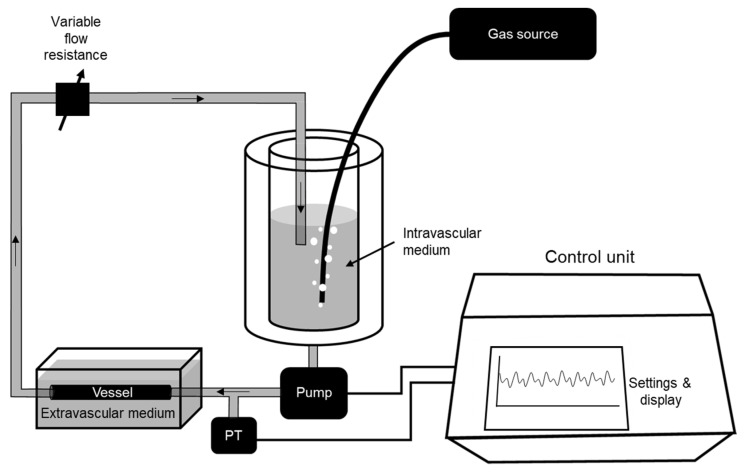
Diagram of the experimental setting. Please see explanation in the text.

The experimental setting developed consists of two chambers and an electronically controlled system to circulate an intravascular medium through the vessel (Figure 1). One of the chambers is devoted to hold the vessel and to keep it submerged in an external medium. The other chamber is a reservoir for the intravascular medium that is continuously bubbled with a gas, having predefined O_2_ and CO_2_ concentrations to achieve the desired intravascular conditions: either normal gas concentrations, or hypo/hyperoxia and/or hypo/hypercapnia. Both chambers were made by 3D printed customized pieces which can be reproduced using any conventional 3D printer by using the driving files freely available in the Supplementary File “Technical_Description.zip”.

The circulation system consists of a conventional car windshield wiper pump (TSP022, 12 V, 2 A, up to 1.5 L/min) that injects pressurized intravascular medium from this reservoir to the vessel. The pump is digitally controlled (Arduino Mega 2560) in real time to reproduce the target intravascular pressure using the actual pressure measured at the inlet of the vessel by means of a disposable pressure transducer for clinically measuring blood pressure (Transpac IT, ICU Medical; technical description and data at https://pdf.medicalexpo.com/pdf/icu-medical/transpac-it/119492-179463.html#open821370, accessed on 2 June 2021) (Figure 1). Calibration of the employed pressure measuring setting (including transducer and electronics) against a reference Hg column was linear within ±2% up to 180 mmHg. The target waveform for intravascular pressure P(t) was generated according to:

P(t) = P_m_ + a_1_ × cos(2π·f_c_ ·t) + a_2_ × cos(4π·f_c_·t + θ) (1)

where t is time, P_m_ represents the mean pressure, f_c_ is the cardiac frequency, a_1_ and a_2_ are the amplitudes of the fundamental and the first harmonic of the waveform, and θ is the phase of the first cardiac harmonic [31].

For any given applied pressure, the value of flow through the vessel was controlled by modifying the resistance of a fluid resistor placed downstream between the outlet of the vessel and the passive return inlet of the fluid reservoir. Modifying the flow (Q) of an incompressible liquid with dynamic Newtonian viscosity (μ) circulating in stationary laminar regime through a rigid-wall cylindrical vessel of a known internal radius (R) allows to control the shear stress (τ) at the vessel wall (endothelium). Indeed, according to the Hagen–Poiseuille equation:

τ = 8 × μ × Q/(πR^3^) (2)

All the electronic circuitry included in the setting was placed in a customized 3D printed enclosure containing the pressure sensor conditioning circuit, including a low-pass filter, the Arduino micro-controller and a 5-inch TFT LCD display with a resistive touch panel (ER-TFTM050-3, 800 × 480 RGB dots). The custom-made code loaded into the Arduino allows the user to modify the pressure waveform parameters (frequency and peak systolic and diastolic pressure) in real time by touching the panel, and to continuously observe the actual time course of pressure.

The retail cost of all electronic and mechanical components to build the setting was EUR ≈ 250. All the technical information and detailed circuit schematics and the controller code required to build this ventilator are available for release under free terms following the open-source hardware approach in the Supplementary File “Technical_Description.zip”.

After sensor calibration and testing of the electro-mechanic and digital components of the setting using silicone tubes with dimensions mimicking vessels, the experimental setting performance was subjected to a proof-of-concept test using an ex vivo preparation of aorta from a rabbit sacrificed in a food-industry slaughterhouse. The abdominal aorta was isolated from other tissues and excised carefully to avoid damage. The vessel was washed with phosphate buffered saline (PBS) to remove blood and other biological remains. An aorta section of 5.5 cm in length was placed into the setting and subjected to a pressure waveform (Equation (1)) with 120 and 80 mmHg of systolic and diastolic pressures, respectively and a frequency of 60 cycles/min. The downstream resistor was set to circulate a 120 mL/min flow of PBS (as measured from the volume circulated for a certain time period). To achieve this flow for such pressure, the downstream resistor consisted of a silicone tube (3 mm of internal diameter, 40 cm in length) plus a hypodermic needle (18G × 1 1/2″).

A conventional cellphone placed 25 cm on top of the vessel allowed to record the changes in vessel diameter as internal pressure was varied, therefore, characterizing vessel mechanics through its pressure–diameter relationship which allowed to derive different stiffness parameters.

## 3. Results

**Figure 2:**
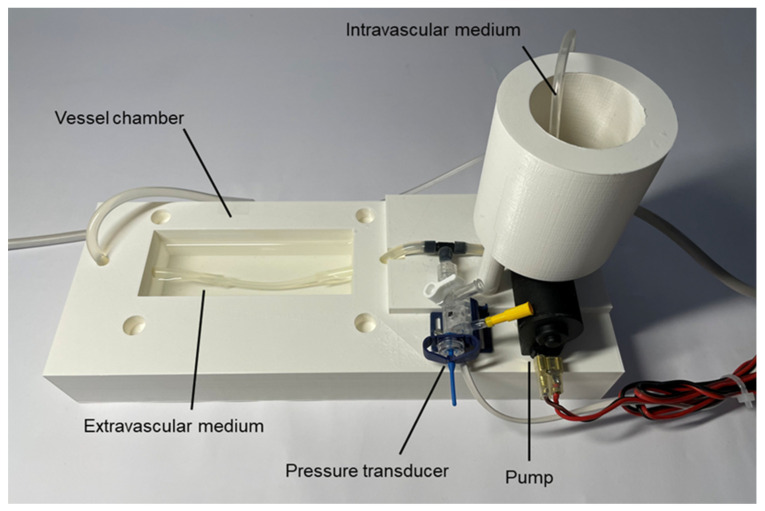
Experimental setting chambers, pressure transducer and pump: vessel chamber, intravascular medium chamber, pump and pressure transducer. See text for explanation.

Figure 2 shows an image of the 3D printed chambers of the setting, including the pump and pressure sensor. In this figure the vessel in the chamber was replaced by a silicone tuning with dimensions similar to a rabbit aorta. The hypodermic needle being a part of the downstream resistor is not visible, since it was placed inside the cylindrical chamber for the intravascular medium. The pressure transducer is at the inlet of the vessel. The pump takes the medium from the base of the cylindrical chamber and directs the medium to the vessel. For the sake of image clarity, the tube bubbling gas into the medium in the cylindrical chamber is not shown. Please see more views of the experimental setting as well as layouts of its pieces in the Technical_Data_and_User_Manual document included in the Supplementary File “Technical_Description.zip”.

**Figure 3:**
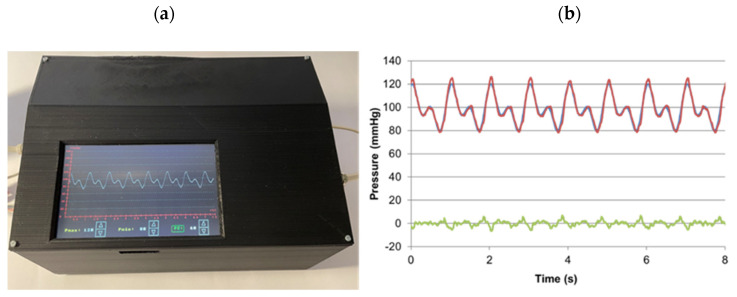
Pressure waveform applied by the setting to an ex vivo rabbit aorta section. (**a**): image taken from the setting controller; (**b**): figure showing the excellent performance of the pressure control system—actual pressure (red), target pressure (blue) and difference between these signals (green).

Figure 3a depicts the real time pressure measured at the aorta inlet as observed in the device display, also showing the setting controls to modify the parameter values. In Figure 3b, this signal is compared with the target pressure waveform, showing minor difference, hence, providing evidence of the good performance of the control system. The average difference (±SD) along the cycle was only 2.18 ± 1.56 mmHg. Similar good results were obtained when frequency was increased to 120 cycles/min and pressures up to 180 mmHg (data not shown).

The mean shear stress at the endothelial layer of the aorta was determined by Equation (2) by considering that the mean external radius along the cycle was 3.72 mm (ranging 3.49–3.96 from diastolic to systolic peaks). Assuming that the aorta wall thickness was 0.3 mm, the mean internal radius was estimated as 3.42 mm, which is a value consistent with published values of rabbit aorta diameter (e.g., 6.2 ± 1.1 mm in [32]). According to Equation (2), assuming a PBS kinetic viscosity of 0.0105 dyn·s/cm^2^ at room temperature and taking into account that flow was 120 mL/min, wall mean shear stress was estimated to be τ = 6.32 dyn/cm^2^, in agreement with published values for rabbit aorta shear stress (in the range 6–10 dyn/cm^2^ [33,34]).

**Figure 4:**
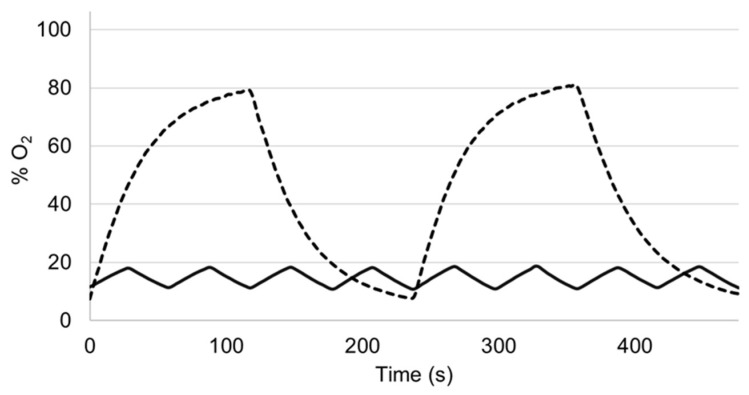
Intermittent hypoxia in the intravascular medium induced by cyclically bubbling gas with different O_2_ concentration into the experimental setting reservoir containing intravascular medium (see text for explanation).

Figure 4 shows examples of how alternatively bubbling gases with a different concentration can subject the endothelium to well-controlled intermittent hypoxia/hyperoxia. For the sake of illustration, these oxygenation signals were measured within the vessel with an auxiliary O_2_ sensor (OXR50; PyroScience, Aachen, Germany) which is not required for the normal function of the setting. In one of the examples (Figure 4), intravascular O_2_ partial pressure mimics low-amplitude high frequency intermittent hypoxemia corresponding to a patient with severe apnea–hypopnea (60 apneas/h) [11]. Figure 4 features the case where high-amplitude, low-frequency intermittent hyperoxia has been employed in studies of potential therapy [35] or to investigate the modulation of ventilatory response and control [36,37].

**Figure 5:**
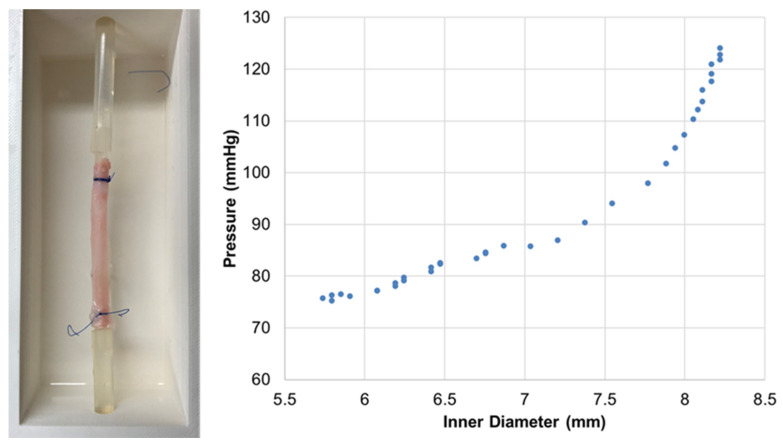
**Left**: Example of one of top view images of the ex vivo rabbit aorta in the setting chamber employed to measure its pressure–diameter relationship; **Right**: Intravascular pressure vs. diameter measured in the rabbit aorta.

Figure 5 shows the measured elastic properties of the rabbit aorta segment, showing the typical nonlinear relationship between pressure and diameter (effective stiffness increasing with pressure) [25,38]. Data from Figure 5 provides an elastance (E) of ≈550 mmHg when computed by using a conventional index: E = ΔP/((D − D_0_)/D_0_)), where ΔP is the pressure difference corresponding to diameter D and to a reference diameter D_0_ (which was taken as the one corresponding to 100 mmHg). Although not directly comparable because of different experimental conditions (e.g., sample biological conditioning, baseline tension, static vs. dynamic), the E value derived from Figure 5 is close to the ≈600 mmHg and ≈515 mmHg (69 kPa) reported when measuring the stiffness of ex vivo mouse and rabbit aortas, respectively [24,38].

## 4. Discussion

The novel experimental approach presented herein allows the application of the following stimuli to an ex vivo vessel preparation: (a) a realistic intravascular pressure waveform defined by the user; (b) a shear stress in the endothelial layer since, in addition to the pressure waveform, the flow through the vessel can be controlled by the user; (c) conditions of hypo/hyperoxia and hypo/hypercapnia in the intravascular circulating medium. Interestingly, these stimuli can be applied alone or in different combinations to study possible synergistic or antagonistic effects on the endothelium. Therefore, the modulating effects on cellular and molecular endothelial function of any biomarkers and drugs under test can be studied under the realistic mechanical constraints and blood gases levels characterizing different pathologies.

The specific experimental setting described in this paper and in the accompanying online technical documentation can be easily modified if required by the user. First, the setting is intended for carrying out experiments at room temperature or at physiological temperature when placed inside a thermostatic chamber: either a conventional culture chamber or, to have an autonomous setting, a customized enclosure adapted to the setting size and equipped with any low-cost general purpose electrical heater with thermostat. Second, the intravascular pressure waveform can be modified, for instance by adding more harmonics, by simply changing the lines corresponding to Equation (1) in the code provided as Supplementary Materials. Third, the diameter of the intravascular medium can be reduced to both decrease the medium volume and, thus, its cost, which is particularly relevant when applying expensive biomarkers or drugs to investigate the endothelium, and to facilitate a faster O_2_-CO_2_ concentration transition in the bubbled intravascular medium. Fourth, the desired shear stress experienced by the vessel endothelium can be easily modified by changing the downstream resistance. Indeed, using hypodermic needles of different size may provide the main adjustment of resistance and modifying the length/diameter of the tubing can provide its fine tuning. The resulting shear stress is estimated from the effective vessel radius and viscosity of the circulating medium (PBS, plasma or blood) at the experiment temperature and assuming that the conditions required by the Hagen–Poiseuille equation are met.

A remarkable feature of the experimental setting presented herein, which is based on similar basic principles as in conventional settings [23,24,38,39], is that it was constructed by using on-the-shelf general purpose electronic (e.g., Arduino, display, disposable pressure sensor) and mechanical (pump from the car industry) components, and thanks to the fact that any widely available conventional 3D printer allows to build the chambers and enclosures at a cost (EUR ≈250) which is by far lower than the one corresponding to commercially available settings with similar functionality. It is noteworthy that the detailed description and files provided in the open-source Supplementary Information may allow for the experimental setting to be built by any electronic technician or engineering student. In fact, the device described here was built by a biomedical engineering student (author A.O.) as her final degree project. Therefore, the open-source hardware approach makes it possible for scientific devices, such as the present ex vivo setting, to be built by persons who are in the academic environment (e.g., schools of engineering) of biomedical labs, thereby facilitating research.

## 5. Conclusions

This work describes an experimental setting for subjecting ex vivo vessels to different pathophysiological conditions reproducing the in vivo vessel environment (e.g., blood pressure oscillations, shear stress, blood gases partial pressure). The main feature of this experimental setting is that it can be easily built at a low cost from the open-source detailed information provided. Accordingly, the technical information provided here may facilitate the study of ex vivo vessel samples for better understanding how the endothelium, with or without drug treatments, responds to physical stimuli characterizing prevalent diseases.

## Data Availability

Data are provided in the online Supplementary Files accompanying this article.

## Supplementary Materials

The following are available online at https://www.mdpi.com/article/10.3390/life11070671/s1, File: Technical_Description.zip.
