# Supplementary material for: Experimental Setting for Applying Mechanical Stimuli to Study the Endothelial Response of Ex Vivo Vessels under Realistic Pathophysiological Environments
Source: Life (Basel). 2021 Jul 8;11(7):671. doi: 10.3390/life11070671 (PMC8306098; doi:10.3390/life11070671)
Supplement: Supplementary file 1 [file life-11-00671-s001.zip › Technical_Description/Technical_Data_and_User_Manual.pdf]

OPEN SOURCE HARDWARE PUBLICATION

TECHNICAL DESCRIPTION

life

MDPI

Article

Experimental setting for applying mechanical stimuli to study the endothelial response of *ex-vivo* vessels under realistic path-ophysiological environments.

Ana Osuna[1], Anna Ulldemolins[1], Hector Sanz-Fraile[1], Jorge Otero[1,2], Núria Farré[3,4,5], Ramon Farré[1,2,6], Isaac Almendros[1,2,6,*]

DISCLAIMER

This document provides technical information on the specific device prototype described in the publication indicated above.

Using components different from (although similar to) the ones described here may require technical adjustments or adaptations and therefore the final performance of the resulting device must be specifically assessed.

The authors of this document are not responsible for the use of the information contained herein nor for any device built using such information.

LICENSE:

This device is released under CERN Open Hardware License (OHL) v1.2 and all software and documentation under GNU General Public License (GPL) v3.0.

1. Driving code

Source code in file: CODE_Arduino.ino (included in the same zip folder where this pdf file is located).

2. Schematics of the circuit

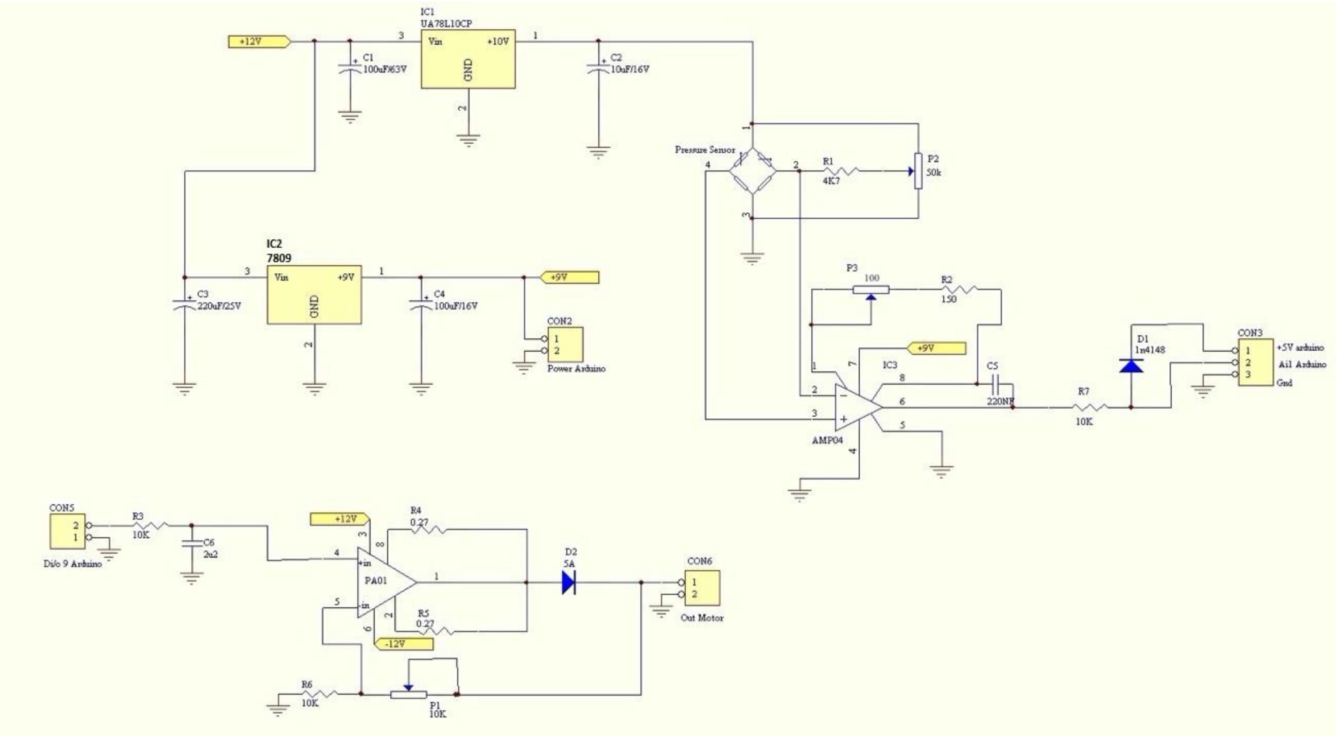

3. PCB board layout

(actual size: 81mmx61mm)

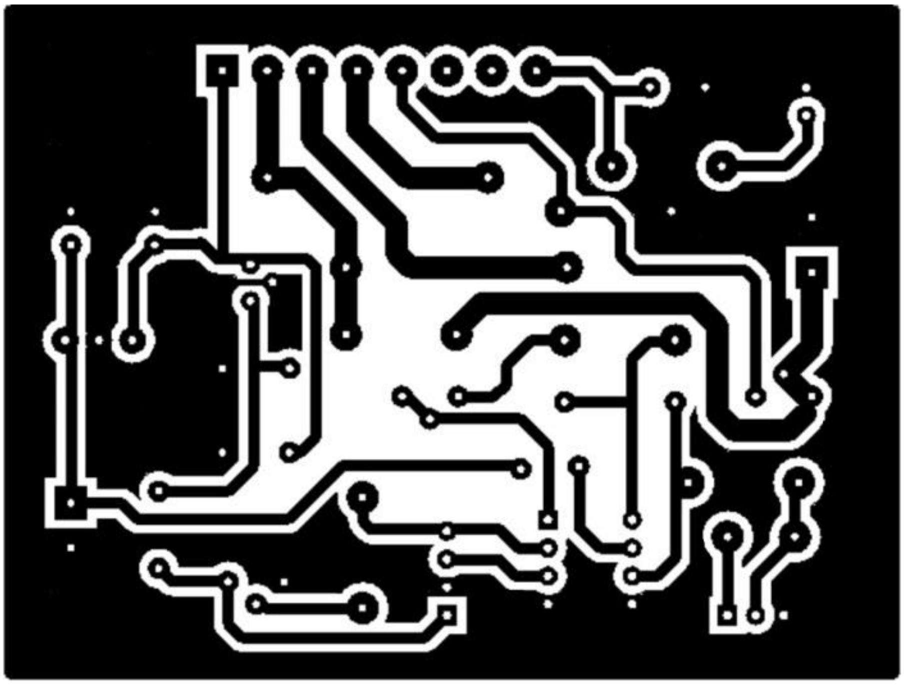

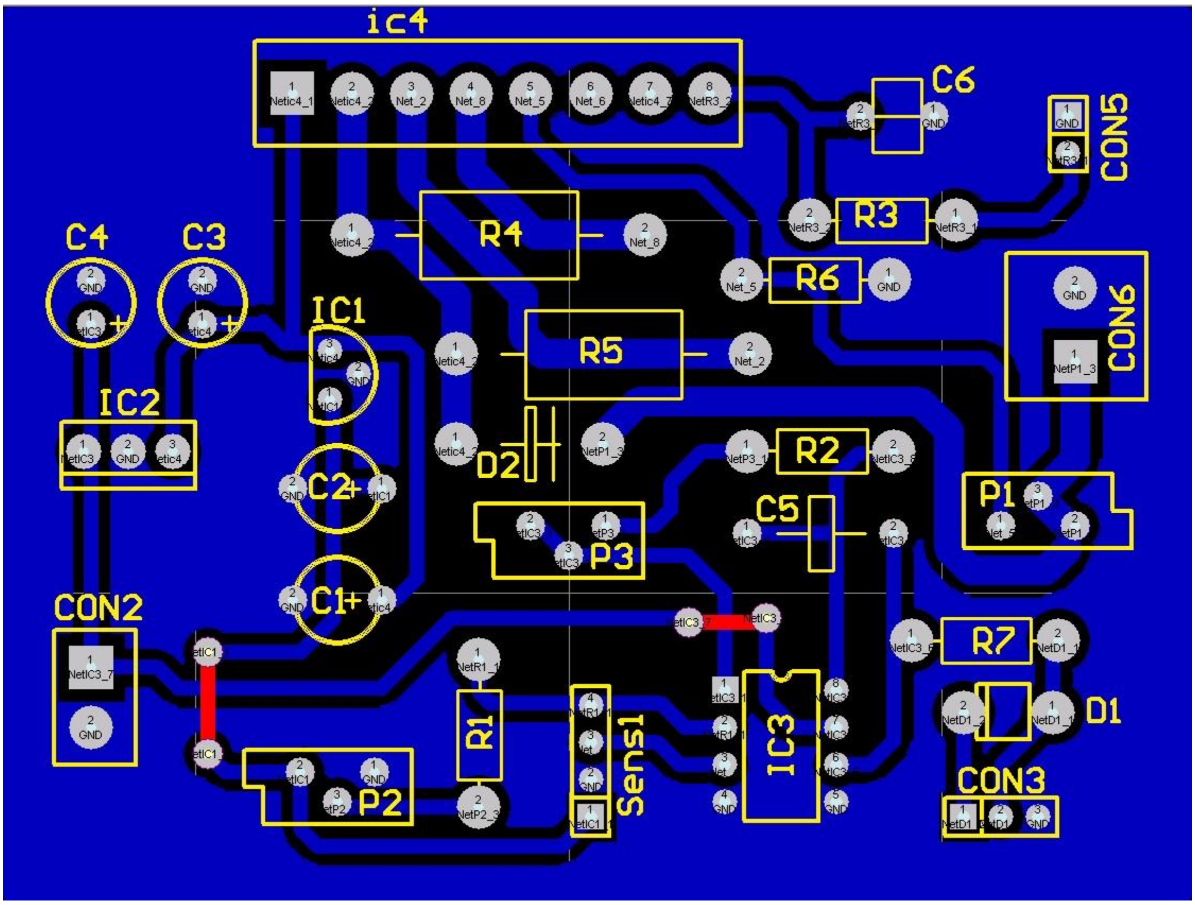

4. List of components

PCB:

- 2 linear voltage regulators: 1x78L10, 1x 7809 (radiator needed)
- Capacitors: 2x 100µF (radial electrolytic), 1x10 µF (radial electrolytic), 1x220 µF (radial electrolytic), 1x220nF (MKT), 1x2,2µF (MKT)
- Resistors: 1x100kΩ (1/4W,5%), 3x10kΩ (1/4W,5%), 2x 0,27Ω (2W), 1x150Ω (1/4W,5%), 1x4,7kΩ (1/4W,5%).
- Potentiometers: 1x10kΩ, 1x50 kΩ, 1x100Ω (ref 3296W)
- Diodes: 1x Signal diode 1n4148, 1x Power diode of 5A.
- Instrumentation amplifier: 1x AMP04
- Integrated power amplifier: 1x PA01
- Microcontroller board Arduino Mega 2560

External:

- TFT LCD display with a resistive touch panel (ER-TFTM050-3, 800x480 RGB dots)
- TFT01 Mega Shield v 1.0 (adapter LCD TFT01 for Arduino MEGA)
- WWP10 (also named TSP022) windshield wiper pump
- Transpac™ IT Integrated Pressure Transducer: sensitivity of 5µV/V/mmHg
- Radiator
- Thermal silicone
- Mica electrical insulator
- Silicone Conduits: Internal radius=3mm, external radius=6mm, length= 40cm
- Hypodermic needle: radius=1.2mm, length=40 mm

Switching power supply ±12V 60W

5. Connections

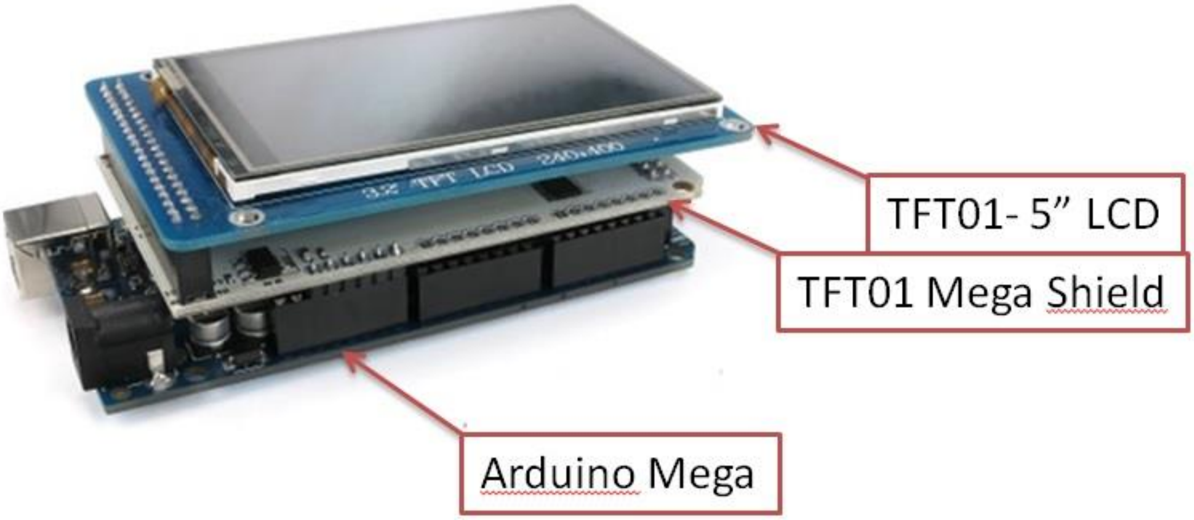

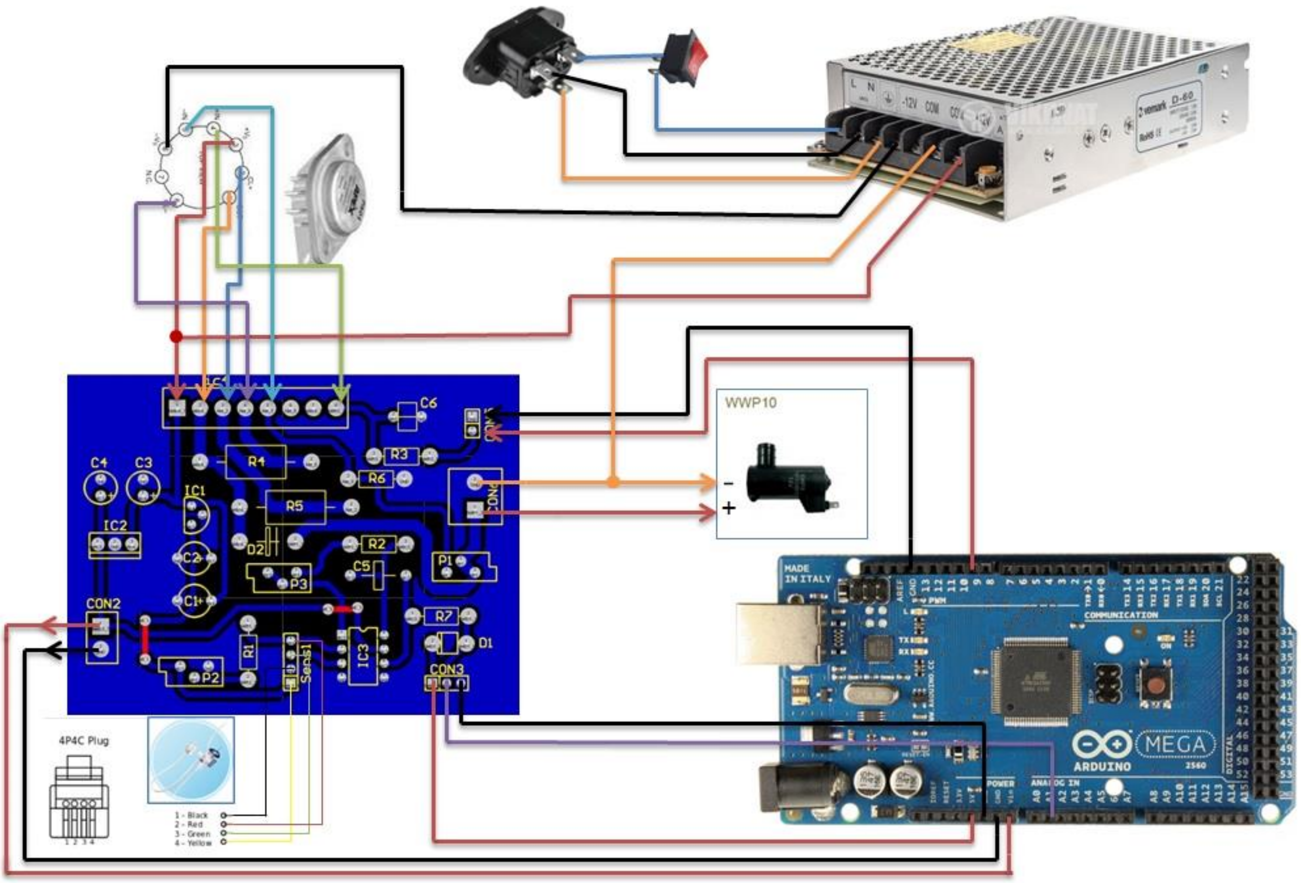

6. Pump

This table was obtained from the provider portfolio (September, 2019): Washer Pump Listing, WIPAC

WWP10

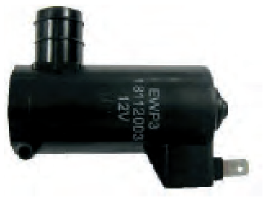

Type:	Direct Fit	Replaces OE6140949
Voltage:	12v	
Elec. Connection:	Lucar	Ford Escort 80-86
Working Current:	2.6A	Ford Fiesta 77-83
Flow Rate (ML/Min)	1500	Ford Scorpio 85-89
Discharge Pressure:	180Kpa	Honda Accord 85-93
No. Outlets	Single	

7. Enclosure by 3D printer

Source code in files: 3D-file1.stl, 3D-file2.stl, 3D-file3.stl, 3D-file4 (included in the same zip folder where this pdf file is located).

- Electronics housing

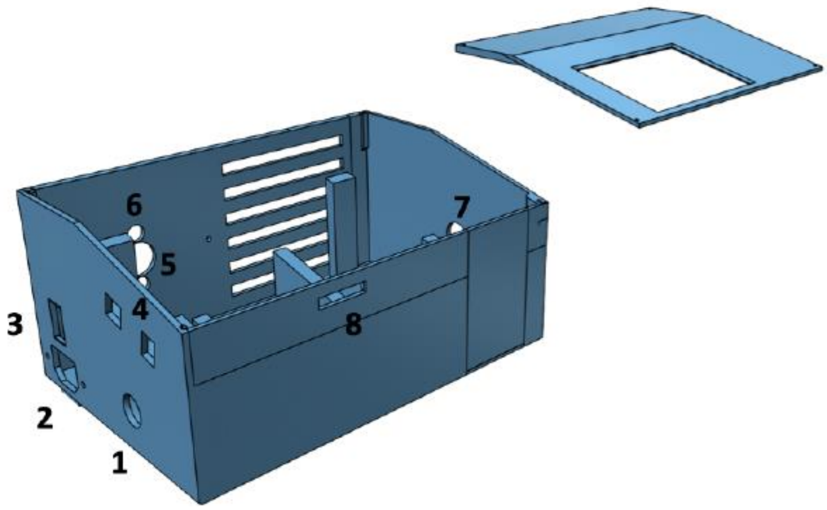

1. Pump connection
2. Electrical network connection
3. Switch
4. Arduino connections
5. PA01
6. Radiator fixation
7. Pressure transducer connection
8. SD entrance

- Media container

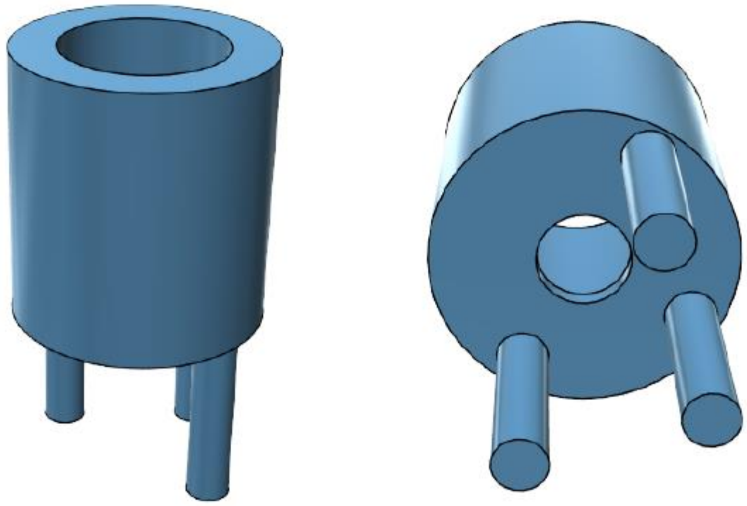

- Bioreactor support

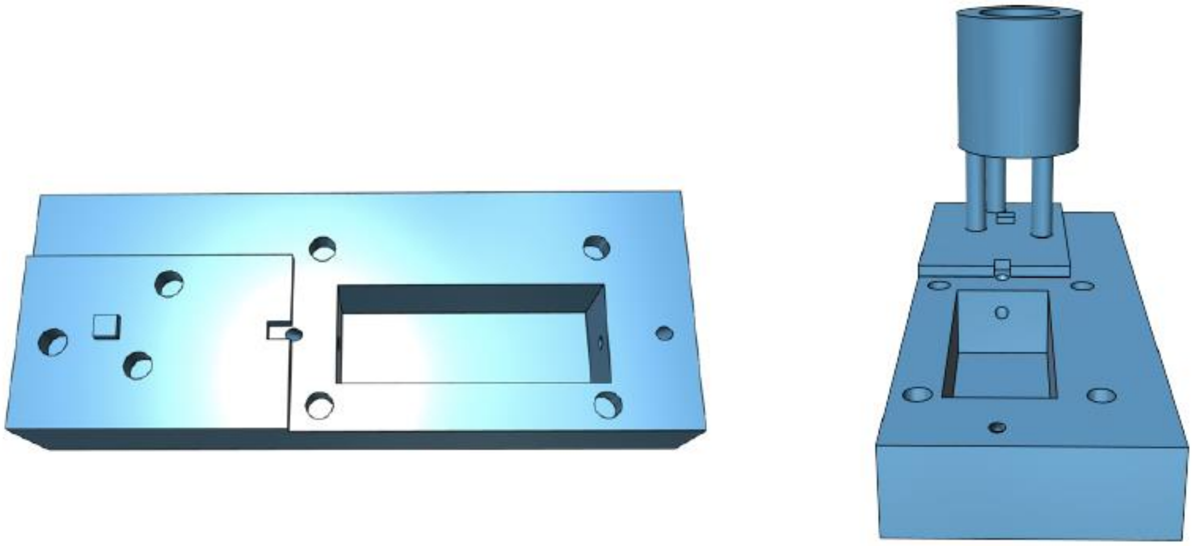

8. Fast user manual

USER'S GUIDE

BIOREACTOR FOR REPRODUCING VASCULAR PULSATILE RADIAL STRETCH AND LONGITUDINAL FLOW-INDUCED SHEAR STRESS PROTOTYPE

Device capable to reproduce both the pulsatile vessel wall cyclic stretch and the shear stress in the wall surface of human arteries. The mechanical properties of some different vessel models and the shear stress induced by mimicked blood flow can be characterized using this prototype. The different parts of the system and its setting description are explained below.

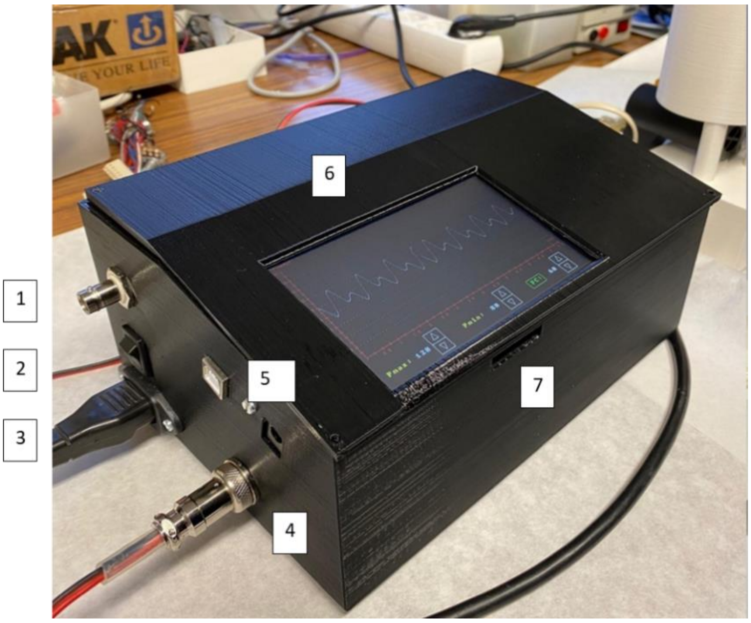

Figure 1. *Lateral view of the electronics of the system.*

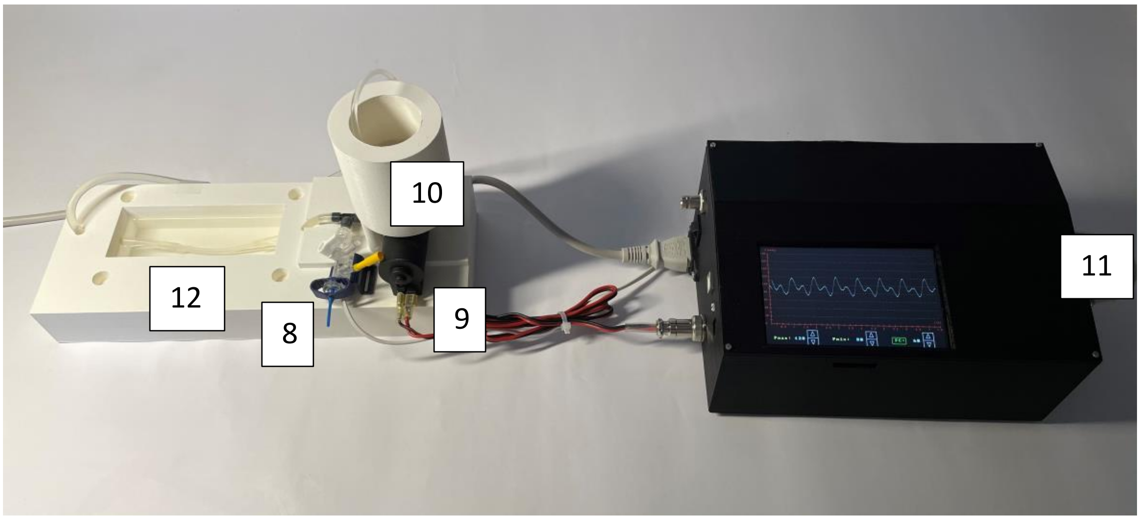

Figure 2. *Top view of all the bioreactor.*

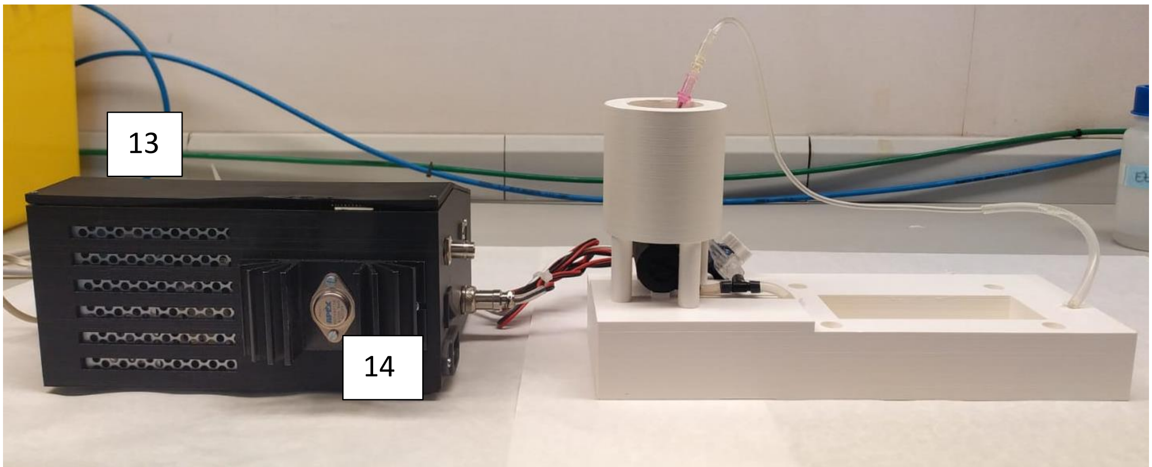

***Figure 3**. Rear view of the bioreactor.*

***Table 1**. Bioreactor's parts summary*

Number	Device feature	Description
1	Signal generator connection	Connection used when a signal generation is required
2	Switch	Turning on or off the device.
3	Electrical network connection	Connection through which the device is powered.
4	Pump connection	The pump is powered and controlled by the system through this connection.
5	Arduino connections	Connection in case the Arduino needs to be reprogramed.
6	5-inch TFT LCD display	This display allows the user to see the real-time pressure wave and to modify the parameters by using the touch screen.
7	SD entrance	If the user wants to register some data in an SD card, it can be inserted through this entrance. However, this feature is not included in the current program.
8	Pressure transducer	This section has been specifically designed for the correct accommodation of the pressure transducer.

9	Pump	This section has been specifically designed for the correct accommodation of the pump.
10	Medium container	In this container, the medium flowing through the circuit is stored.
11	Pressure transducer connection	This is the connection through which the pressure transducer is connected to the system.
12	Bath recipient	In this space, it is placed the tissue engineered vascular graft (TEVG).
13	Ventilation slots	Through these slots, the electronic circuit is refrigerated.
14	Radiator	This radiator is used to avoid the heating produced by the dissipation of power of the voltage regulators.

SETTING DESCRIPTION FOR APPLYING THE PRESSURE PULSE

1. Upload the Arduino board with the desired program through the Arduino connections.
2. Connect the pump to the system through the pump connection.
3. Connect the pressure transducer to the system through pressure transducer connection.
4. Place your TEVG into the bath and connect it to the system.
5. Connect the device to the power source through electrical network connection.
6. Turn the switch on. The touch screen will show an initial message: "Unitat de Biofísica, Universitat de Barcelona".
7. After a few seconds, the real-time signal will appear on the screen showing how the pulse is applied to the vessel.
8. To change the pressure parameters, press the up and down narrows shown in the screen accordingly referred as "Pmax" and "Pmin".
9. To change the frequency rate, press the up and down narrows shown in the screen accordingly. It is necessary to click within the green button to reestablish the frequency and start the new pulse.
10. Data is extracted by the "Serial Monitor" tool offered by Arduino.
11. Use the switch to turn off the system.

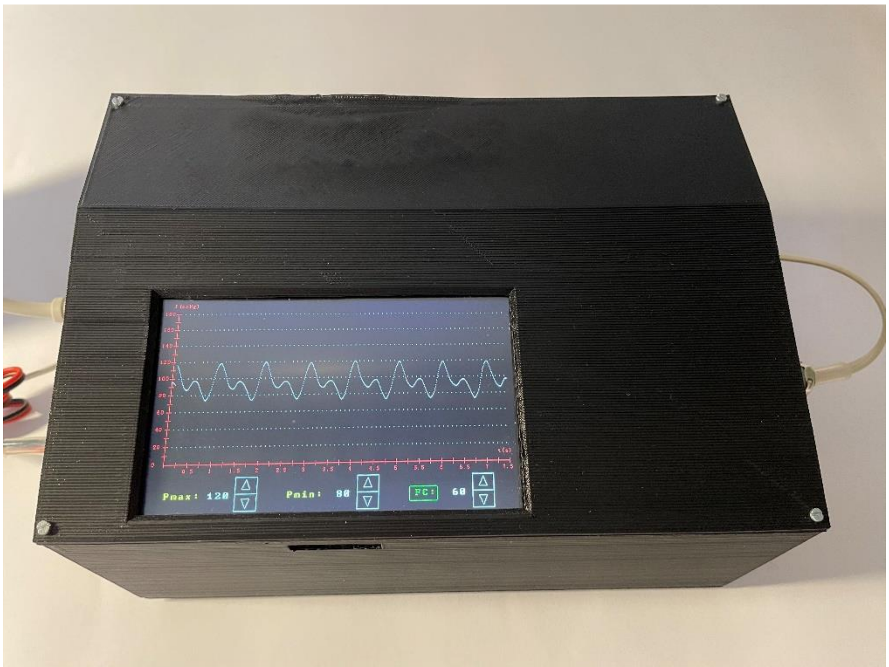

Figure 4. *Real time signal of the pressure applied to the vessel.*

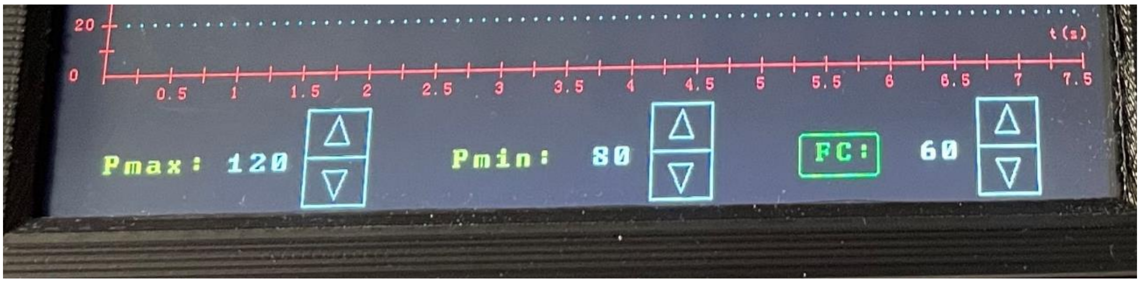

Figure 5. *Touching panel for change the pressure parameters and the frequency rate.*
